# Phenotype to genotype in *Neurospora crassa*: Association of the *scumbo* phenotype with mutations in the gene encoding ceramide C9-methyltransferase

**DOI:** 10.1016/j.crmicr.2022.100117

**Published:** 2022-02-19

**Authors:** Erin L. Bredeweg, Kevin McCluskey, Scott E. Baker

**Affiliations:** a Functional and Systems Biology Group, Environmental Molecular Sciences Division, Pacific Northwest National Laboratory, Richland, WA, USA; b Fungal Genetics Stock Center, Kansas State University, USA; c DOE Joint BioEnergy Institute, Emeryville, CA, USA

**Keywords:** Neurospora, Scumbo, Ceramide C9-methyltransferase, Morphology, Fungi, Fungal genomics, Fungal genetics, Fast-forward genetics

## Abstract

Using a legacy of genetic mutants of *Neurospora crassa*, paired with resequencing efforts through JGI, we have identified the gene responsible for the ‘scumbo’ mutant. This early morphological mutant was described as “Irregular flat, spreading growth with knobby protrusions and abnormal conidiation, but no free conidia. Mycelium usually appears yellowish rather than orange. Female fertile.” (Perkins, Radford et al. 2000). Our further investigation has found new insights into the identity and associated functions of scumbo as a ceramide C9 methyltransferase, previously annotated as “similar to cyclopropane-fatty-acyl-phospholipidsynthase”, encoded by the gene NCU07859. This enzyme performs a fungal-specific methyl modification of glycosyl-ceramides and has implications for membrane homeostasis and hyphal polarity in filamentous fungi.

## Highlights

• Genome resequencing of classical genetic mutant strains associated gene with phenotype.
• *Neurospora crassa* gene *scumbo* encodes a ceramide C9-methyltransferase.
• Deletion of *Neurospora crassa* ceramide C9-methyltransferase encoding gene is viable.

## Graphical abstract

Graphical abstract

**Figure:**
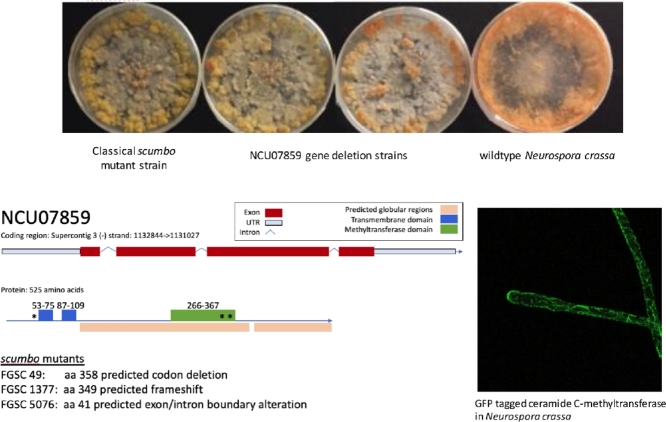

## 1 Introduction

Morphology is an easily scored phenotype for filamentous fungi. As such, a significant number of morphological mutants have been identified, genetically mapped, and characterized in the model fungus, *Neurospora crassa* (Garnjobst and Tatum 1967). When Beadle and Tatum performed screens to understand the biochemical genetics of *N. crassa*, they also isolated a series of mutants called*visible* (*vis*), a catchall name for genes with morphological phenotypes (Houlahan, Beadle et al. 1949). Morphological mutants from mutagenesis screens may have defects that result in a disruption to polar hyphal growth and/or branching, cytoskeletal structure or function, or secretory genes (Seiler and Plamann 2003, McCluskey, Wiest et al. 2011, Lara-Rojas, Bartnicki-Garcia et al. 2016). For example, *N. crassa smco-9*, or semi-colonial-9 was thought to be caused by altered “branching enzyme” expression (Abramsky and Tatum 1976) while alpha-COP coatamers were found to be essential for polarized growth in *Aspergillus nidulans* (Whittaker, Lunness et al. 1999). Additional determinants of differentiation and morphology may be associated with handling or modifying membrane lipids, such as flippases which were recently characterized in *A. nidulans* (Schultzhaus, Cunningham et al. 2019), or the effects of phospholipid methyltransferase, as seen in mutants of *Pestalotiopsis microspore* (Akhberdi, Zhang et al. 2018).

Some of the earliest discovered morphological mutants were used in early studies for genetically locating auxotrophic biochemical genes. One of the *vis* alleles (5801 - FGSC strain 49) was later mapped and renamed *scumbo* (*sc*) (Barratt, Newmeyer et al. 1954). *Neurospora crassa* morphological mutants are classified into several different groups based on their features (Garnjobst and Tatum 1967). *scumbo* is classified a semi-colonial mutation, described as a category of “mutants that begin growth on agar as a small colony and sooner or later produce a flare of wild-type-appearing hyphae, with or without conidia.”(Garnjobst and Tatum 1967). The *scumbo* phenotype mutant is easily recognized by its low spreading growth, knobby protrusions, and abnormal conidiation (Barratt, Newmeyer et al. 1954). Fertile as a female and residing within Linkage Group III, *scumbo* made an excellent marker that was utilized to genetically map *leucineless* or *leu-1* (Houlahan, Beadle et al. 1949). Analysis by scanning electron microscopy confirmed that the *scumbo* mutation resulted in “abnormal conidiophores'' (Springer and Yanofsky 1989). While multiple studies have shown that morphological mutations could lead to defects in cell wall composition, the nature of many of these genes remained elusive. For example, *sc* was shown to have differences in cell wall sugar composition compared to wildtype (Cardemil and Pincheira 1979). A single allele of a modifier of scumbo, *mod-sc* was isolated and mapped to LGIV (Hsu 1963). A recent screen of *Neurospora* wild type isolates revealed several morphologies of conidiophore development (Krach, Wu et al. 2020) but did not reveal phenotypes similar to the extreme *scumbo* defect.

Phenotypes tracked in morphological mutants also include osmosensitivity and resistance to antifungal compounds, such as dicarboximides, e.g., vinclozolin. A linkage between these phenotypes has identified a shared mode of action in which dicarboximides affect osmotic regulation and membrane function which also is regulated through the histidine kinase *os-1* pathway (Grindle 1984, Schumacher, Enderlin et al. 1997, Fujimura, Ochiai et al. 2000, Ochiai, Fujimura et al. 2001, Cui, Beever et al. 2002, Oshima, Fujimura et al. 2002). Resistance to vinclozolin results in osmosensitivity. Morphological mutations in *N. crassa* genes with phenotypes similar to *sc* have been identified, including the classical morphological mutants, *smco-8* and *smco-9* (Grindle and Temple 1983).

Despite their utility for genetic mapping, a significant number of morphological mutations in *N. crassa* remain “anonymous” or unassociated with a physical locus in the genome. The use of next generation sequencing technologies to rapidly identify mutations and associate them with phenotypes has been called “fast forward genetics” (Darby and Hall 2008, Schneeberger and Weigel 2011). This strategy has great potential to link genes with biological function in fungal systems (Baker 2009, Baker and Bredeweg 2016, Baker 2018). In previous work we used genetic map data combined with a population of single nucleotide variants from several *N. crassa* strains to identify candidate mutations to associate with anonymous mutant genes (McCluskey, Wiest et al. 2011). Another approach is to sequence the genome of multiple strains that each carry independently isolated alleles of the target mutant gene that have been shown to be alleles by complementation analysis in genetic crosses (Garnjobst and Tatum 1967, Perkins, Radford et al. 2000). These different alleles may contain different mutations in a single gene. In this study we describe our use genome resequencing to facilitate the rapid identification of the morphological mutants of *N. crassa, sc* as well as phenotypically related mutants, *smco-8* and *smco-9*. Identification of the causal gene for the *scumbo* phenotype by sequencing allowed us to test the hypothesis that NCU07859 is *scumbo* by gene knockout. Testing and confirmation of historically collected mutants merges longstanding observations with gene function, and morphological structures and functional inter-dependencies.

## 2 Materials and Methods

### 2.1 Genomic DNA preparation and sequencing

Strains of *N. crassa* were maintained and DNA extracted as described previously (Gabriel, Thieme et al. 2021). To summarize, *N. crassa* strains shown in Table 1, were preserved on anhydrous silica gel (Perkins 1962) in the Fungal Genetics Stock Center collection with minimal cycles of preservation and regrowth, some for as long as 50 years without passage. Genotypes were routinely tested as part of best practices (Wiest, Schnittker et al. 2012). Cultures for DNA extraction were prepared by first sprinkling a few grains of silica gel containing preserved conidia onto agar solidified Vogel's minimal medium (Vogels 1956). After approximately one week, conidia were inoculated into 10 ml Vogel's minimal medium in a 50 ml disposable conical test tube using a heat sterilized inoculating loop. These cultures were incubated at room temperature (18 – 22 ⁰C) with shaking at approximately 100 cycles per minute for 2 to 3 days. A pad of mycelia was removed from the culture with a sterile wooden applicator stick and blotted dry on sterilized paper towels. 90 – 120 mg of tissue was weighed and transferred to a macerating tube and DNA extraction was carried out according to the instructions provided by the manufacturer (ZR Fungal/Bacterial DNA MiniPrep, Zymo Research, Irvine, CA). Tissue maceration was carried out using a Mini-Beadbeater-16 (BioSpec Products, Bartlesville, OK) for 30 seconds and set to half of maximum power. DNA quality was assessed by separating a sample in an agarose gel using standard practices and by spectrophotometric analysis using a NanoDrop microvolume spectrophotometer (Thermo Scientific, Wilmington, DE). DNA was held at - 20⁰ until being sent on dry ice to the US DOE JGI for sequencing and analysis as previously described (McCluskey, Wiest et al. 2011, Reilly, Kim et al. 2018). Briefly, the JGI generated DNA libraries for paired-end sequencing method using MiSeq 2  ×  150 bp (∼ 30 × coverage) or HiSeq 2  ×  100 bp (∼ 100 × coverage). Genome sequences from each strain were compared to the reference genome for identification of SNPs and indels using BCFtools and GATK tools (McKenna, Hanna et al. 2010, Li 2011) and subsequently compared against each other to identify mutations unique to each strain compared against an aggregate of strain resequencing data from the *Neurospora* resequencing project (doi: 10.25585/1487571).

**Table 1:** Mutant strains

**Gene**	**FSGC strain(s)**	**NCBI BioProject accession**	**Mutation**	**Location and Annotation**
*scumbo*	49	PRJNA251072	Supercontig_3:1131635; GACGTGC→G; two codons lost: EHV341D	NCU07859 - ceramide C9-methyltransferase
	1377	PRJNA249825	Supercontig_3:1131614 TCCC→ TCC; Frameshift	
	5076	PRJNA250153	Supercontig_3:1132617; C→T, Supercontig_3:1132618; T→C; splice acceptor mutation	
*semicolonial-8*	8247	PRJNA346042	Supercontig_4:516041, C→G; Start codon lost, M1→I1	NCU08811 - ADP-ribosylation factor GTPase activating protein (ARF GAP)
*semicolonial-9*	9414	PRJNA250936	Supercontig_4: 4537010, C→T; missense E343K	NCU07280 - serine/threonine-protein kinase stk-50/gad8
*mod(sc)*	1162	PRJNA249820	Supercontig_4:3476049, A→G; splice acceptor site	NCU04404 - coatomer beta subunit
	1163	PRJNA249873		

### 2.2 Isolation and characterization of homokaryon deletion strains

FGSC 13992 (NCU07859 heterokaryon) was used as the maternal strain with FGSC 2489, 74-OR23-1VA as the fertilizing strain in a cross. Resulting ascospores were isolated and tested for hygromycin resistance, the marker used to delete genes in the deletion collection. To test the genotype of the isolated ascospore homokaryons, strains were grown on hygromycin B (100 μg/mL) in comparison to wild type and the classic *scumbo* mutant FGSC 49. Inoculum for these tests was generated by growing test strains on Vogel's medium agar. Uniform agar blocks (3mm square) of hyphae were cut from the colony edge and placed on plates (6 cm) containing 8 mL of Vogel's glucose agar alone or containing drug as indicated. Imaging was after 1 week of strain outgrowth.

### 2.3 Osmotic challenge plate assay

Conidia or hyphal fragments were cultivated on agar solidified Vogel's minimal medium with 1.5% glucose for 30 hours. Uniform blocks (3 millimeters per side) containing hyphae were cut and placed on new small plates (6 cm) containing 6 mL the same medium with or without the osmotic agent listed (.25 M NaCl, 1 M NaCl, 3% Ethanol). Imaging was conducted after 120 hours of growth at room temperature.

### 2.4 Strain generation and microscopy for GFP localization of ceramide C9-methyltransferase

To generate a C-terminal transcriptional fusion to observe localization of the scumbo enzyme, we designed primers to amplify 1 kb regions up and downstream of the stop codon of NCU07859 using q5 polymerase (New England Biolabs) reaction conditions (5’ fragment from OEB 392, 5’-GACCCCAACAAGGAGGAGA-3’ and OEB393, 5’-CCTCCGCCTCCGCCTCCGCCGCCTCCGCCGTTGGCAGGGACAGTGGG-3’; 3’ fragment from OEB394, 5’-TGCTATACGAAGTTATGGATCCGAGCTCGAAGCGGCAAAGGACGACA-3’ and OEB395, 5’-CCATTAGGATTCGAGGCAGA-3’). These 5’ and 3’ fragments were integrated by overlap PCR with a plasmid fragment (pGFP∷hph∷loxP) containing GFP and *hph* (amplified from the plasmid with primers OEB59, 5’-CGAGCTCGGATCCATAACTTCGTATAGCA-3’ and OKP31, 5’-GGCGGAGGCGGCGGAGGCGGAGGCGGAGG-3’). We used primers to create an overlap in the middle of *hph* (OEB57, 5’-GTGCTTTCAGCTTCGATGTAGG-3’, paired with OEB392; and OEB58, 5’-AGAAGATGTTGGCGACCTCG-3’ paired with OEB395), while leaving individual 5’ and 3’ PCR fragments with an incomplete *hph* gene to ensure homologous recombination (Honda and Selker 2009). Initial fragments were amplified using 32 cycles, followed by gel purification. Overlap PCRs were run 5 cycles with only template DNA fragments (5’ fragment or 3’ fragment each with GFP-*hph* fragment), followed by addition of primers and a further 32 cycles. These PCR fragments were gel purified and added in equimolar amounts to *Neurospora crassa* conidia (FGSC9718, delta mus-51::bar+, mat a) for electroporation (Colot, Park et al. 2006). Details of PCR primer placement and overlap construction are included in Supplemental Fig. SB. Colonies were picked to individual slants, and screened for GFP signal by fluorescence microscopy as noted below. A GFP-positive strain from this transformation TEB146.1 was crossed to the wild type strain 74-OR23-1VA (FGSC2489, mat A) to obtain the homokaryon strain FEB369.

Conidia of strain FEB369 were grown on Vogel's agar in a 100 mL Erlenmeyer flask, with 5 minutes of sunlight exposure at 24 and 28 hours to support conidiation before collection at 7 days. Conidia were collected and stored in sterile distilled water at 5C. For confocal microscopy, conidia were extensively diluted, and grown in 2 mL of 1% glucose Vogel's liquid media in 35 mm dishes, with amendments. Amendments include 0.25 M NaCl or 3% ethanol. For imaging, a small section of dispersed tissue was transferred onto a glass slide in 5-10 μl of media and covered for imaging with a glass coverslip. Imaging was performed on the Leica 710 confocal laser scanning microscope with a 100X oil immersion lens.

### 2.5 Conidia germination microscopy

Conidia grown as above were collected by wetted wooden stick or from powdery tissue released on plate lid and suspended in sterile distilled water. A 50 μl aliquot of this suspension was added to 150 μl 2% Vogel's liquid medium in a 1.7 mL Eppendorf and incubated without shaking at room temperature for 6-7 hours. Results of this experiment are presented in Supplemental figure SA.

## 3 Results

### 3.1 Associating scumbo with a physical locus

**Fig. 1:**
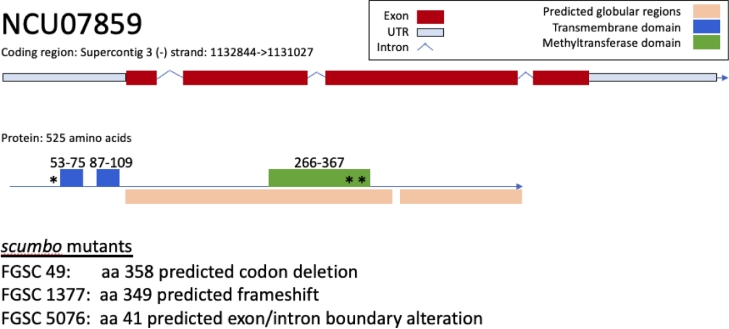
**Gene model with exon structures and placement of scumbo mutant features in FGSC***N****eurospora* strains**. Secondary structure and SMART/pfam domains were predicted by eukaryotic linear motif (Kumar, Gouw et al. 2019). NCU07859 is 525 amino acids long and features two transmembrane domains (spanning amino acids 53-75 and 87-109) and a methyltransferase domain (amino acids 266-367) identified from SMART/Pfam domain families. Resequencing of FGSC49 found a codon deletion in the methyltransferase domain. Resequencing of FGSC1377 found a frameshift in the methyltransferase domain. Resequencing of FGSC5076 found an altered exon/intron boundary at amino acid 41 upstream of the transmembrane domain. Each mutation location is marked by asterisk.

We sequenced the entire genome of three strains of *N. crassa* each carrying a unique allele of *scumbo* (Table 1). In each strain we found a mutation in the open reading frame designated NCU07859 which, by orthology with other fungi, has been hypothesized to encode a ceramide C9-methyltransferase (Table 1). This gene has also been annotated as ​​cyclopropane fatty acyl phospholipid synthase, and as *gsl-9* (FungiDB). This enzyme, only found in fungi, adds a methyl-group to the C9 carbon of a ceramide hydrocarbon chain. The first mutant allele of *scumbo* isolated, 5801, contained a six bp deletion causing a change at glutamic acid-histidine-valine (EHV) to a single aspartic acid (D) at residue 341. These residues are conserved across fungal orthologues and occur in a methyltransferase domain. Allele R2386 contains a frameshift while allele R2503 has a mutation in the intron splice site acceptor (Fig. 1).

**Fig. 2:**
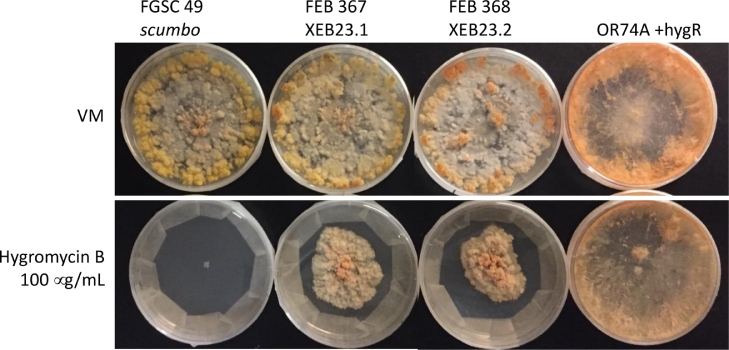
**Generation and testing of *scumbo* homokaryons susceptibility to hygromycin B.** To establish the segregation of the scumbo phenotype, we crossed FGSC13992, identified as a heterokaryon NCU07859 from the knockout collection, to OR74A (FGSC2489). FGSC13992 was used as the maternal strain. Ascospores were collected and germinated by heat shock, isolated to individual slants, and tested for mating type using spot plates. XEB23.1 is *mat A*, XEB23.2 is *mat a*. To test susceptibility to hygromycin B, we first grew hyphal tissue on small agar plates, cutting 3 mm squares as a means of equal inoculum. Plates were cultured for 7 days before imaging.

After identification of the locus consistent with the *scumbo* phenotype, strains carrying targeted gene deletion mutations at NCU07589 were obtained from the *Neurospora* gene deletion collection (Colot, Park et al. 2006, Dunlap, Borkovich et al. 2007, Park, Colot et al. 2011). The knockouts for NCU07589 were deposited as heterokaryons. We undertook the isolation of homokaryons using a genetic cross. FGSC 13992 (NCU07859 heterokaryon) was used as the maternal strain with FGSC 2489, 74-OR23-1VA as the fertilizing strain in a cross. Resulting ascospores were isolated and tested for hygromycin resistance, the marker used to delete genes in the deletion collection. Multiple ascospore progeny from each mating type were isolated. The morphological phenotype of homokaryons derived from the deletion collection strain mirrors that of the classical *scumbo* allele containing strains (Fig. 2).

While identifying *scumbo*-associated genes and morphological mutants, we also re-sequenced strains for *smco-8, smco-9*, and a modifier of the *scumbo* defect, *mod(sc)* as part of a larger resequencing project (doi: 10.25585/1487571). (Table 1). We identified mutations in ADP-ribosylation factor GTPase activating protein (ARF GAP) (NCU08811), serine/threonine-protein kinase gad8 (NCU07280) and coatomer beta subunit (NCU04404), respectively.

### 3.2 Phenotyping

**Fig. 3:**
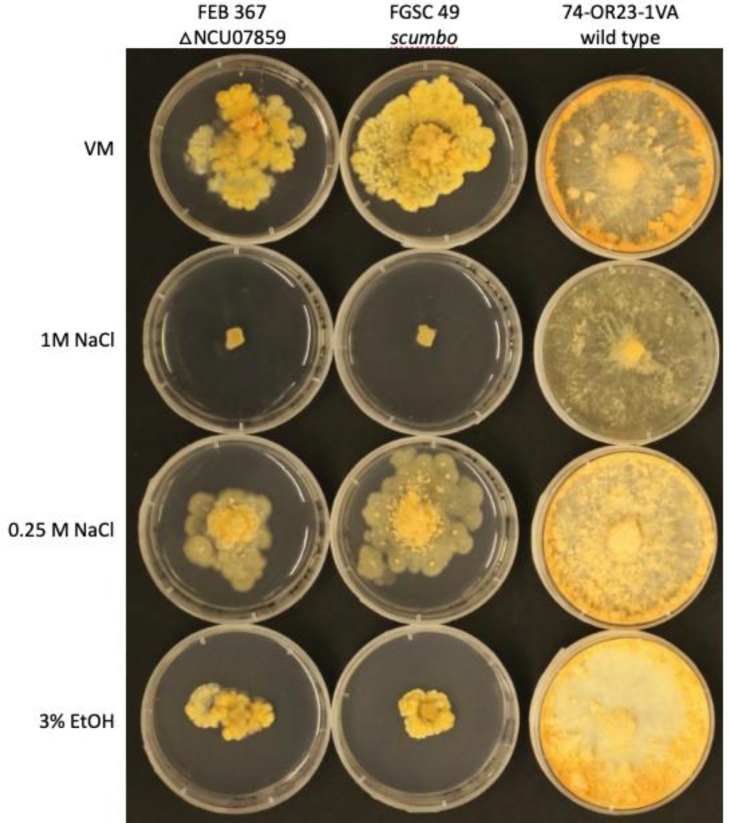
**Plate growth with osmotic stressors**. To assay growth on stress conditions, conidia or hyphal fragments were grown on plates containing VM for 24-36 hours. 3 mm agar blocks were cut and placed on the center of each plate and sealed using micropore surgical tape. Plates were imaged after 5 days of growth. VM:Vogel's medium with 1.5% sucrose.

In many fungi, C9 methylation of ceramide is a key step in fungal synthesis of glucosylceramide and galactosylceramide and is implicated in pathogenicity [in *Cryptococcus neoformans* (Singh, Wang et al. 2012, Raj, Nazemidashtarjandi et al. 2017), *Pichia pastoris* (Ternes, Sperling et al. 2006), and *Fusarium graminearum* (Ramamoorthy, Cahoon et al. 2009)], implicated in pH stress response and linear growth, [in *Aspergillus nidulans* (Levery, Momany et al. 2002) and *Candida albicans* (Oura and Kajiwara 2010)]. It has also been implicated in cell differentiation, with ceramide monohexosides (CMHs) containing this modification being found in hyphal, but not conidial tissue of *Pseudallescheria boydii* (Pinto, Rodrigues et al. 2002). This may be consistent with the phenotypes seen in *N. crassa*, of aconidial growth (inhibited hyphal elongation, and non-linear asci formation). Because of the role ceramide C9-methyltransferase and its product play in the development of plasma membrane we tested wildtype and mutant strain response to a variety of osmotic stresses, which are known to be dependent on plasma membrane stability (e.g. (Ianutsevich, Danilova et al. 2016)) (Fig. 3). Our tests indicated susceptibility of scumbo to hyphal extension under osmotic stressors of ethanol and salt.

### 3.3 Localization of ceramide C9-methyltransferase by microscopy

**Fig. 4:**
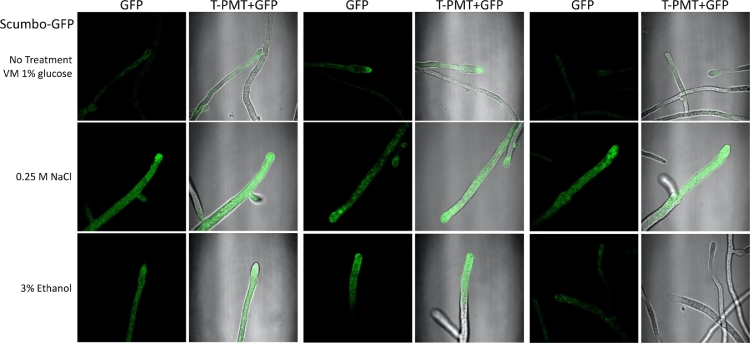
**Microscopy of Scumbo-GFP.** A strain with NCU07859 transcriptionally fused with a C-terminal GFP tag, imaged under different conditions. Conidia were cultured in 2 mL of media with the indicated supplements for 16 hours with no agitation at room temperature. Tissue was then transferred to a microscope slide with 5-10 μl of media and covered with a coverslip for image capture on a Leica 710 confocal microscope. Three representative paired images are shown, normalized to a common GFP range using Fiji. At the left of each pair is the GFP channel, right is the overlay of GFP with photomultiplier tube (T-PMT) transmission.

**Fig. 5:**
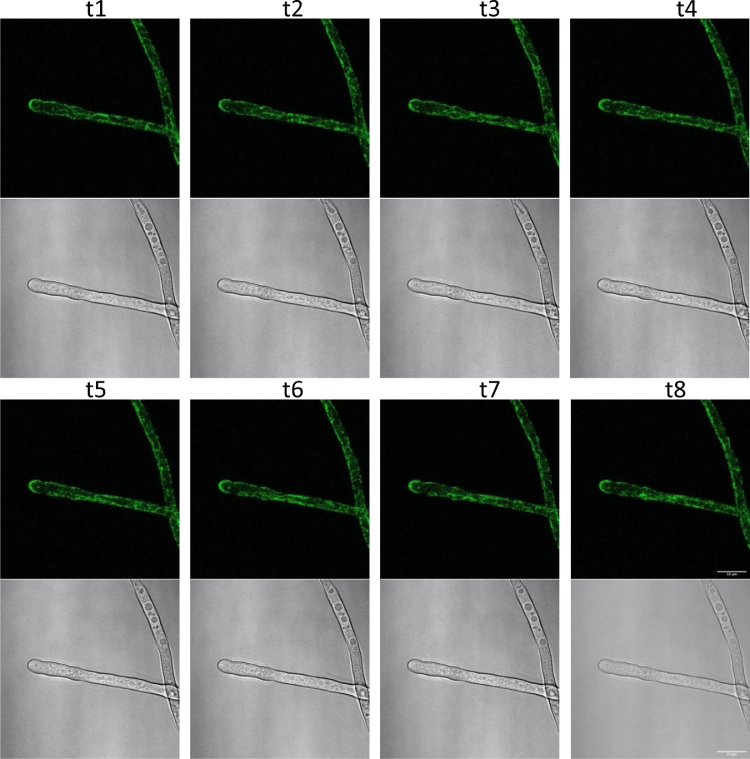
**Scumbo-GFP in a time-series**. Images were taken of the same hyphae of Scumbo-GFP, showing accumulation at the end of the hyphae's Spitzenkörper, or collection of vesicles at the hyphal tip. Scumbo signal fluctuates along the endomembrane system. Each timepoint represents a capture over approximately 2 minutes by scanning confocal microscope. Scale bar is 10 μm in t8.

We sought to explore the localization of ceramide C9-methyltransferase in *N. crassa* and constructed a C-terminal GFP tagged ceramide C9-methyltransferase strain. Localization of this enzyme has not been previously shown directly in filamentous fungi. After transformation with a C-terminal GFP cassette in a NHEJ-defective strain (FGSC9718, Δ mus-51::bar+, mat a), the GFP positive (screened by microscopy) strain was backcrossed to 74-OR23-1VA (XEB 37). The resulting ascospores were heat shocked, picked to individual agar slants, and screened for GFP. The resulting strain (XEB 37.1, FEB369) was morphologically indistinguishable from wildtype indicating that the addition of GFP did not noticeably alter enzyme function. Interestingly, the localization of the GFP signal changes over time, with respect to the hyphal tip, and osmotic stressor (Figs. 4, 5). NCU07859 contains 2 transmembrane domains (amino acids 53-75, and 87-109) allowing membrane association.

## 4 Discussion

A prodigious number of classical genetic mutants remain “anonymous”; the low cost and high coverage generated by current sequencing methods makes it tractable to simply re-sequence these strains to identify the mutation responsible. Indeed, resequencing *N. crassa* has been particularly successful, associating genes with mutant phenotypes. Here, we describe the identification of NCU07859 which encodes a ceramide C9-methyltransferase as the classical *Neurospora crassa* morphological mutant, *scumbo*. Supporting this identification is the fact that multiple alleles of *scumbo* were sequenced and each had mutations in NCU07859. In addition, we generated homokaryotic strains from the *N. crassa* gene deletion project heterokaryon strain. These strains with deleted NCU07859 phenocopied the classical *scumbo* strains.

The strain from the *N. crassa* gene deletion project deposited at the Fungal Genetics Stock Center is a heterokaryon which led to the hypothesis that NCU07859 is an essential gene in *N. crassa*. Detailed phenotypic analysis was done on the heterokaryon by Huber et al (Huber, Oemer et al. 2019). Research from *Fusarium* and *Aspergillus nidulans* have concluded that ceramide C9-methyltransferase is essential. However, in some yeasts where the gene encoding the ceramide C9-methyltransferase has been deleted growth is impacted but not eliminated. By crossing the *N. crassa* heterokaryon with wildtype we generated homokaryotic mutants which are morphologically identical to classically genetically derived *scumbo* strains. The existence of homokaryotic *scumbo* classical mutants and generation of homokaryotic deletion strains indicate that NCU07859 is not an essential gene.

Sensing of osmotic stress occurs at the cell membrane and initiates a significant cell signaling response. Moreover, the cell plasma membrane composition is remodeled at both the lipid and protein levels. In *Neurospora*, the two-component histidine kinase *nik1*(*os-1)-rrg1* responds to osmotic stress triggering a downstream response through the *hog1* MAP kinase cascade (*os-2, os-4, os-5*)(Jones, Greer-Phillips et al. 2007). Evidence from a variety of species across the fungal kingdom indicates that the histidine kinase sensors are organized by lipid rafts and that sphingolipids play an important role in their formation (Singh, Wang et al. 2012, Tanigawa, Kihara et al. 2012, Fernandes, de Castro et al. 2016, Raj, Nazemidashtarjandi et al. 2017). Yeast and filamentous fungal osmosensor sensor and signaling machinery are dependent upon glucosylceramides for proper function. We hypothesize that the *sc* phenotype is due in part to disruption of these rafts caused by disruption to the glucosylceramide biosynthetic pathway.

As cells remodel to deal with osmotic stress, anterograde and retrograde vesicle transport pathways play an obvious role, and both are strongly influenced by sphingolipids. The similar phenotypes of vinclozolin resistance and osmotic sensitivity of *sc, smco-8* and *smco-9* imply that proteins encoded by these genes may be involved in the biological processes involved in both osmotic stress sensitivity and dicarboximide resistance. Our results indicate that *smco-8* (NCU08811) encodes an ADP-ribosylation factor GTPase-activating protein (Arf1GAP) orthologous to *S. cerevisiae* GLO3 and *mod(sc)* (NCU04404) encodes a coatomer-beta orthologous to *S. cerevisiae* SEC26. Multiple studies in *Saccharomyces* have linked GLO3 and SEC26 (DeRegis, Rahl et al. 2008, Schindler, Rodriguez et al. 2009). Arf1GAPs are linked to COP(I) coatamer vesicle formation during priming and assembly of the coat protein on a membrane surface (Beck, Ravet et al. 2009). The protein encoded by *smco-9* (NCU07280 also called *stk-50*) is an ACG kinase that is activated by TOR kinase, with homologs in *S. cerevisiae* (YPK1) (Roelants, Torrance et al. 2002) and *S. pombe* (Gad8) (Martín, Portantier et al. 2017). YPK1 activity is required for plasma membrane lipid and protein homeostasis, cell wall integrity, and endocytosis suggesting a common mechanism for colonial defect formation in the fundamental structure membranes of these colonial phenotypes.

We localized SC to the endomembrane system and the Spitzenkörper in growing hyphae. This localization is consistent with the hypothesis that the plasma membrane is dynamic and characterized by the ability to rapidly remodel in response to stress. The localization phenotype is also consistent with proteins modifying lipids at the golgi apparatus and associated organelle membrane structures. Our observations were done early in growth (i.e. within 20 hours of germination) (Figs. 4, 5, and SB). Additional expression patterns are suggested for older tissue: the gene is expressed and protein accumulates at the cell periphery where modifications take place (Kasuga and Glass 2008).

Plants and animals do not have orthologs of the ceramide C9-methyltransferase making C9-methylation a unique modification which distinguishes fungal sphingolipids from plant and animal lipids (Ternes, Sperling et al. 2006). The presence of C9-methylation has also been used in defenses from other organisms, such as plant and insect defensins against *P. pastoris* and *C. albicans* (Thevissen, Warnecke et al. 2004) but is less important in *F. graminearum* susceptibility (Ramamoorthy, Cahoon et al. 2009). The *N. crassa* heterokaryon for NCU07859 does show an altered lipid profile and was tested for antimicrobial protein susceptibility (Huber, Oemer et al. 2019). It is unknown if the *scumbo* deletion homokaryon would perform differently. Yeast pleiotropic drug resistance pathways and regulation have also shown linkage to levels of sphingolipids and biosynthetic enzyme expression (Hallstrom, Lambert et al. 2001, Kolaczkowski, Kolaczkowska et al. 2004). Taken together this modification is a gateway to fungal-specific drug development and structural regulation of stress responses.

Mutants at the locus NCU07859 have a phenotype classically described as poor conidial development. Our observations during osmotic challenge plate assays indicated formation of powdery conidia-containing tissue, which suggests that *scumbo* doesn't inhibit conidial development under all conditions. For example, conidiation was associated with physical disruption of hyphae, as occurred during agar block cutting. Conidial development was also not observed under some stress conditions (e.g., EtOH stress, Fig. 3). Though functional sphingolipid C9-methyltransferase activity may be important for conidial germination or subsequent processes, we did not observe any defects in the ability of *scumbo* conidia to begin hyphal elongation. Other studies have noted NCU07859 is strongly upregulated during progression through the four stages conidial germination (Wang, Miguel-Rojas et al. 2019).

Extensive data on *Neurospora* population genomics and transcriptomics are available at FungiDB (Stajich, Harris et al. 2012, Basenko, Pulman et al. 2018). These data show that there is only one transcript from NCU07859 and that there are no non-synonymous or nonsense mutations at this locus. This strain population includes reference strains, mutants, and a group of 48 strains from wild populations (Ellison, Hall et al. 2011). Expression of NCU07859 was characterized and is shown to vary during sexual development (Coradetti, Craig et al. 2012, Wang, Lopez-Giraldez et al. 2014), during growth on cellulose versus sucrose (Coradetti, Craig et al. 2012), and among strains from the wild type population (Ellison, Hall et al. 2011).

In sum, identification of scumbo as ceramide C9-methyltransferase emphasizes the importance of lipid membranes in stress resistance, signaling and morphology. The striking morphology and phenotypes of the scumbo mutant suggests that this uniquely fungal lipid modification affects cell physiology and gene expression. Transcript level changes stemming from altered levels of modified glucosylceramide will be a topic for future work.

## CRediT authorship contribution statement

**Erin L. Bredeweg:** Conceptualization, Writing – original draft, Investigation. **Kevin McCluskey:** Conceptualization, Investigation, Writing – review & editing. **Scott E. Baker:** Conceptualization, Project administration, Writing – original draft, Investigation.

## Declaration of Competing Interest

The authors declare that they have no known competing financial interests or personal relationships that could have appeared to influence the work reported in this paper.

## Appendix Supplementary materials

Image, application 1
