# Supplementary material for: Phenotype to genotype in Neurospora crassa: Association of the scumbo phenotype with mutations in the gene encoding ceramide C9-methyltransferase
Source: Curr Res Microb Sci. 2022 Feb 19;3:100117. doi: 10.1016/j.crmicr.2022.100117 (PMC9325734; doi:10.1016/j.crmicr.2022.100117)
Supplement: Supplementary file 1 [file mmc1.docx]

**Supplementary figures**


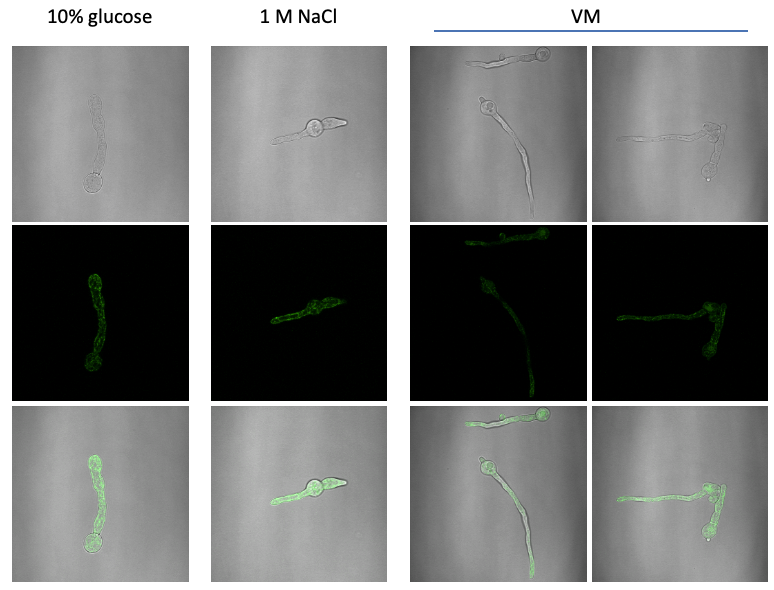


**Supplemental Figure SA. Microscopy of germinated conidia in GFP-tagged *scumbo* strain under different osmotic stressors**

**Supplemental Figure SB. Schematic showing placement of primers for overlap PCR during construction of the scumbo-GFP strain.**  Genomic and plasmid DNA fragments were fused by overlap PCR for transformation into *N. crassa* conidia to generate the transcriptional fusion of scumbo with GFP.
